# RETRACTION: Capsaicin Protects Cardiomyocytes against Anoxia/Reoxygenation Injury via Preventing Mitochondrial Dysfunction Mediated by SIRT1

**DOI:** 10.1155/omcl/9813621

**Published:** 2026-06-02

**Authors:** Oxidative Medicine and Cellular Longevity

RETRACTION: H. He, Y. Zhou, J. Huang, et al., “Capsaicin Protects Cardiomyocytes against Anoxia/Reoxygenation Injury via Preventing Mitochondrial Dysfunction Mediated by SIRT1,” *Oxidative Medicine and Cellular Longevity*, 2017, 1035702, https://doi.org/10.1155/2017/1035702.

The above article, published online on 24 December 2017 in Wiley Online Library (wileyonlinelibrary.com), has been retracted by agreement between the Journal Editor‐in‐Chief, Jeannette Vasquez‐Vivar; and John Wiley & Sons Ltd. The retraction has been agreed due to concerns regarding the Figures, initially reported on PubPeer [1] and raised directly to the publisher by the authors. Further investigation by the Publisher identified additional issues with the figures. A summary of the concerns can be found below:

• In Figure 6a, the two rows of cytosol appear identical. Additionally, when adjusted they appear similar to the Full Length Caspase 3 bands in Figure 8 of [2]
• The top right (A/R) and bottom right (Cap+A/R+Sirtinol) flow cytometry plots in Figure 9a appear identical
• When re‐sized, the ß‐Actin bands shown in Figures 2 and 8b appear highly similar

After assessment of the author’s response and the concerns by the Chief Editor, the results and conclusions can no longer be considered reliable, and the article is therefore retracted.

The authors agree with the retraction.
